# 

**Published:** 1866-05

**Authors:** 


### THE
### CHICAGO
### MEDICAL JOURNAL.

Vol. XXIII. MAY, 1866. No. 5.

CHICAGO:

JAMES BARNET, PRINTER, 191 LAKE STREET.

